# Methyl 2-(2-amino-1,3-thia­zol-4-yl)-2-[(*Z*)-methoxy­carbonyl­methoxy­imino]ethano­ate

**DOI:** 10.1107/S1600536809007661

**Published:** 2009-03-06

**Authors:** Shahzad Sharif, M. Nawaz Tahir, Islam Ullah Khan, Sarfraz Ahmad, Manan Ayub Salariya

**Affiliations:** a Department of Chemistry, Government College University, Lahore, Pakistan; b Department of Physics, University of Sargodha, Sargodha, Pakistan; c Pharmagen Ltd, Lahore 54000, Pakistan

## Abstract

In the mol­ecule of the title compound, C_9_H_11_N_3_O_5_S, the thia­zole ring is oriented at dihedral angles of 87.33 (3) and 87.18 (3)° with respect to the planar (r.m.s. deviations 0.0136 and 0.0139 Å) methyl ester groups. The dihedral angle between the methyl ester groups is 44.20 (3)°. In the crystal structure, inter­molecular N—H⋯N, N—H⋯O and C—H⋯O hydrogen bonds link the mol­ecules along the *a* axis, through *R*_2_^2^(8) and *R*_2_^2^(22) ring motifs, forming infinite two-dimensional polymeric sheets. π–π Contacts between the thia­zole rings [centroid–centroid distance = 3.536 (2) Å] may further stabilize the structure.

## Related literature

For general background, see: Fu *et al.* (2005 ▶); Saprykina *et al.* (2006 ▶). For a related structure, see: Cheng (2007 ▶). For bond-length data, see: Allen *et al.* (1987 ▶). For ring motifs, see: Bernstein *et al.* (1995 ▶).
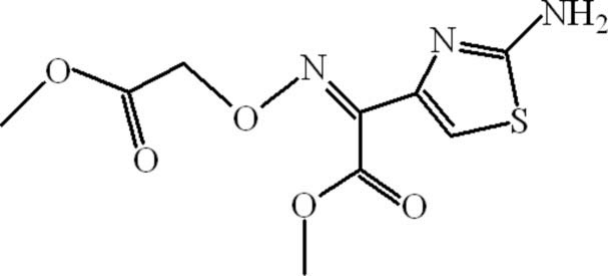


## Experimental

### 

#### Crystal data

C_9_H_11_N_3_O_5_S
*M* *_r_* = 273.27
Monoclinic, 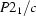

*a* = 12.240 (2) Å
*b* = 5.7500 (8) Å
*c* = 19.887 (3) Å
β = 120.016 (8)°
*V* = 1211.9 (3) Å^3^
*Z* = 4
Mo *K*α radiation
μ = 0.29 mm^−1^
*T* = 296 K
0.22 × 0.08 × 0.06 mm

#### Data collection

Bruker Kappa APEXII CCD area-detector diffractometer
Absorption correction: multi-scan (*SADABS*; Bruker, 2005 ▶) *T*_min_ = 0.975, *T*_max_ = 0.982
10459 measured reflections
2168 independent reflections
1102 reflections with *I* > 2σ(*I*)
*R*_int_ = 0.100

#### Refinement

*R*[*F* ^2^ > 2σ(*F* ^2^)] = 0.051
*wR*(*F* ^2^) = 0.124
*S* = 1.01
2168 reflections
163 parameters
H-atom parameters constrained
Δρ_max_ = 0.24 e Å^−3^
Δρ_min_ = −0.25 e Å^−3^

### 

Data collection: *APEX2* (Bruker, 2007 ▶); cell refinement: *SAINT* (Bruker, 2007 ▶); data reduction: *SAINT*; program(s) used to solve structure: *SHELXS97* (Sheldrick, 2008 ▶); program(s) used to refine structure: *SHELXL97* (Sheldrick, 2008 ▶); molecular graphics: *ORTEP-3 for Windows* (Farrugia, 1997 ▶) and *PLATON* (Spek, 2009 ▶); software used to prepare material for publication: *WinGX* (Farrugia, 1999 ▶) and *PLATON*.

## Supplementary Material

Crystal structure: contains datablocks global, I. DOI: 10.1107/S1600536809007661/hk2639sup1.cif

Structure factors: contains datablocks I. DOI: 10.1107/S1600536809007661/hk2639Isup2.hkl

Additional supplementary materials:  crystallographic information; 3D view; checkCIF report

## Figures and Tables

**Table 1:** Hydrogen-bond geometry (Å, °)

*D*—H⋯*A*	*D*—H	H⋯*A*	*D*⋯*A*	*D*—H⋯*A*
N2—H2*A*⋯N1^i^	0.86	2.22	3.057 (5)	163
N2—H2*B*⋯O5^ii^	0.86	2.27	3.097 (5)	160
C6—H6*A*⋯O3^iii^	0.96	2.53	3.408 (5)	152
C6—H6*C*⋯O4^iv^	0.96	2.51	3.385 (6)	152

Symmetry codes: (i) 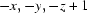; (ii) 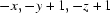; (iii) 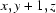; (iv) 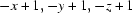.

**Fig. 1.**
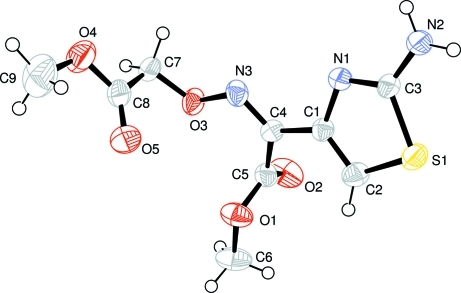
The molecular structure of the title molecule, with the atom-numbering scheme. Displacement ellipsoids are drawn at the 50% probability level.

**Fig. 2.**
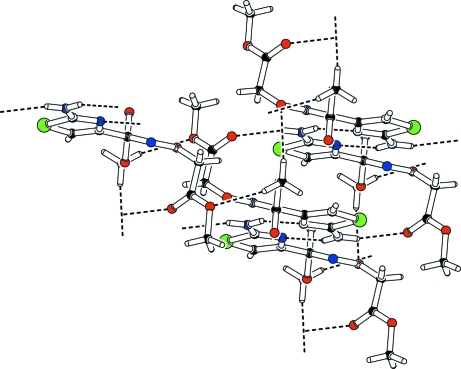
A partial packing diagram of the title compound. Hydrogen bonds are shown as dashed lines.

## supplementary crystallographic information

### Comment

The title compound belongs to thiazole group of organic compounds. Thiazole derivatives have been widely used as intermediates for the syntheses of pharmaceutical active products (Saprykina *et al.*, 2006). 2-Amino thiazole derivatives are the special compounds, which are used for the syntheses of antibiotics (Fu *et al.*, 2005) such as cephalosporins. We report herein the crystal structure of the title compound, (I), which was obtained by alkolysis of mica ester (*S*-2-benzothiazolyl(*Z*)-2-(2-aminothiazole -4-yl)-2-methoxy-carbonylmethoxyiminothioacetate) in methanol.

In the molecule of (I), (Fig. 1), the bond lengths (Allen *et al.*, 1987) and angles are within normal ranges. (I) is different from (*Z*)-(2-amino- thiazol-1-ium-4-yl)-2-(t-butoxycarbonylmethoxyimino)acetate monohydrate, (II) (Cheng, 2007), due to the attachements at carboxylate groups. In (I), ring A (S1/N1/C1-C3) is, of course, planar, and it is oriented with respect to the planar methyl ester moieties (O1/O2/C5/C6) and (O4/O5/C8/C9) at dihedral angles of 87.33 (3) and 87.18 (3) °, respectively, while the dihedral angle between the methyl ester moieties is 44.20 (3)°.

In the crystal structure, intermolecular N-H···N, N-H···O and C-H···O hydrogen bonds (Table 1) link the molecules (Fig. 2), in which they may be effective in the stabilization of the structure. The N-H···N hydrogen bonds link the molecules into dimers by forming the R_2_^2^(8) ring motifs (Bernstein *et al.*, 1995), then N-H···O hydrogen bonds connect the dimers by forming R_2_^2^(22) ring motifs, and C-H···O hydrogen bonds interlink the dimers along the *a* axis forming infinite two-dimensional polymeric sheets. The π-π contact between the thiazole rings, Cg1—Cg1^i^ [symmetry code: (i) -x, 1 - y, 1 - z, where Cg1 is centroid of the ring A (S1/N1/C1-C3)] may further stabilize the structure, with centroid-centroid  distance of 3.536 (2) Å.

### Experimental

For the preparation of the title compound, mica ester, *S*-2-benzo- thiazolyl-(*Z*)-2-(2-aminothiazole-4-yl)-2-methoxy-carbonylmethoxyimino- thioacetate) (1.0 g, 2.195 mmol) was suspended in methanol (10 ml). The suspension was heated, stirred at pH = 6.5 for 15 min. The clear transparent mixture was allowed to cool at room temperature from which light yellow crystals were obtained after 3 d.

### Refinement

H atoms were positioned geometrically, with N-H = 0.86 Å (for NH_2_), C-H = 0.93, 0.96 and 0.97 Å for aromatic, methyl and methylene H, respectively, and constrained to ride on their parent atoms, with U_iso_(H) = xU_eq_(C,N), where x = 1.5 for methyl H and x = 1.2 for all other H atoms.

### Crystal data

**Table:**

C_9_H_11_N_3_O_5_S	*F*(000) = 568
*M**_r_* = 273.27	*D*_x_ = 1.498 Mg m^−^^3^
Monoclinic, *P*2_1_/*c*	Mo *K*α radiation, λ = 0.71073 Å
Hall symbol: -P 2ybc	Cell parameters from 2168 reflections
*a* = 12.240 (2) Å	θ = 2.2–25.2°
*b* = 5.7500 (8) Å	µ = 0.28 mm^−^^1^
*c* = 19.887 (3) Å	*T* = 296 K
β = 120.016 (8)°	Needle, yellow
*V* = 1211.9 (3) Å^3^	0.22 × 0.08 × 0.06 mm
*Z* = 4	

### Data collection

**Table:**

Bruker Kappa APEXII CCD area-detector diffractometer	2168 independent reflections
Radiation source: fine-focus sealed tube	1102 reflections with *I* > 2σ(*I*)
graphite	*R*_int_ = 0.100
Detector resolution: 7.80 pixels mm^-1^	θ_max_ = 25.2°, θ_min_ = 2.2°
ω scans	*h* = −14→14
Absorption correction: multi-scan (*SADABS*; Bruker, 2005)	*k* = −6→6
*T*_min_ = 0.975, *T*_max_ = 0.982	*l* = −23→23
10459 measured reflections	

### Refinement

**Table:**

Refinement on *F*^2^	Primary atom site location: structure-invariant direct methods
Least-squares matrix: full	Secondary atom site location: difference Fourier map
*R*[*F*^2^ > 2σ(*F*^2^)] = 0.051	Hydrogen site location: inferred from neighbouring sites
*wR*(*F*^2^) = 0.124	H-atom parameters constrained
*S* = 1.01	*w* = 1/[σ^2^(*F*_o_^2^) + (0.0434*P*)^2^] where *P* = (*F*_o_^2^ + 2*F*_c_^2^)/3
2168 reflections	(Δ/σ)_max_ < 0.001
163 parameters	Δρ_max_ = 0.24 e Å^−^^3^
0 restraints	Δρ_min_ = −0.25 e Å^−^^3^

### Special details

**Table:**

Geometry. Bond distances, angles *etc*. have been calculated using the rounded fractional coordinates. All su's are estimated from the variances of the (full) variance-covariance matrix. The cell e.s.d.'s are taken into account in the estimation of distances, angles and torsion angles
Refinement. Refinement of *F*^2^ against ALL reflections. The weighted *R*-factor *wR* and goodness of fit *S* are based on *F*^2^, conventional *R*-factors *R* are based on *F*, with *F* set to zero for negative *F*^2^. The threshold expression of *F*^2^ > σ(*F*^2^) is used only for calculating *R*-factors(gt) *etc*. and is not relevant to the choice of reflections for refinement. *R*-factors based on *F*^2^ are statistically about twice as large as those based on *F*, and *R*- factors based on ALL data will be even larger.

### Fractional atomic coordinates and isotropic or equivalent isotropic displacement parameters (Å^2^)

**Table:**

	*x*	*y*	*z*	*U*_iso_*/*U*_eq_	
S1	−0.20130 (10)	0.51478 (17)	0.37288 (6)	0.0437 (4)	
O1	0.2059 (3)	0.7883 (4)	0.38158 (15)	0.0513 (11)	
O2	0.1345 (3)	0.5059 (5)	0.29312 (18)	0.0625 (14)	
O3	0.3512 (2)	0.3538 (4)	0.46873 (15)	0.0426 (10)	
O4	0.5574 (3)	0.3846 (5)	0.67254 (18)	0.0613 (12)	
O5	0.4360 (3)	0.6603 (5)	0.59065 (17)	0.0574 (11)	
N1	−0.0076 (3)	0.2430 (5)	0.43907 (18)	0.0351 (11)	
N2	−0.1795 (3)	0.1177 (5)	0.45036 (18)	0.0469 (14)	
N3	0.2360 (3)	0.3246 (5)	0.46982 (18)	0.0383 (12)	
C1	0.0212 (3)	0.4257 (6)	0.4048 (2)	0.0312 (12)	
C2	−0.0702 (3)	0.5859 (6)	0.3675 (2)	0.0408 (16)	
C3	−0.1223 (4)	0.2676 (6)	0.4263 (2)	0.0348 (12)	
C4	0.1463 (4)	0.4379 (6)	0.4141 (2)	0.0322 (12)	
C5	0.1617 (4)	0.5785 (7)	0.3553 (3)	0.0372 (14)	
C6	0.2126 (4)	0.9446 (7)	0.3265 (3)	0.0620 (19)	
C7	0.4506 (3)	0.2847 (6)	0.5423 (2)	0.0422 (14)	
C8	0.4774 (4)	0.4681 (7)	0.6026 (3)	0.0426 (16)	
C9	0.6022 (5)	0.5459 (9)	0.7365 (3)	0.082 (2)	
H2	−0.06294	0.71649	0.34245	0.0490*	
H2A	−0.14022	−0.00431	0.47613	0.0560*	
H2B	−0.25559	0.14364	0.44005	0.0560*	
H6A	0.24631	1.09153	0.35108	0.0935*	
H6B	0.12955	0.96701	0.28279	0.0935*	
H6C	0.26633	0.87853	0.30921	0.0935*	
H7A	0.52605	0.25699	0.53910	0.0503*	
H7B	0.42814	0.14045	0.55766	0.0503*	
H9A	0.65920	0.46760	0.78391	0.1228*	
H9B	0.53185	0.60529	0.73993	0.1228*	
H9C	0.64524	0.67231	0.72808	0.1228*	

### Atomic displacement parameters (Å^2^)

**Table:**

	*U*^11^	*U*^22^	*U*^33^	*U*^12^	*U*^13^	*U*^23^
S1	0.0355 (6)	0.0453 (6)	0.0495 (8)	0.0083 (5)	0.0206 (6)	0.0116 (5)
O1	0.074 (2)	0.0389 (17)	0.047 (2)	−0.0059 (15)	0.0348 (18)	0.0026 (14)
O2	0.094 (3)	0.0595 (19)	0.043 (2)	−0.0171 (18)	0.0411 (19)	−0.0062 (16)
O3	0.0295 (16)	0.0579 (18)	0.0405 (18)	−0.0004 (13)	0.0175 (14)	0.0102 (14)
O4	0.057 (2)	0.061 (2)	0.045 (2)	0.0107 (16)	0.0098 (18)	0.0004 (16)
O5	0.052 (2)	0.0447 (19)	0.069 (2)	0.0084 (15)	0.0254 (18)	0.0027 (15)
N1	0.033 (2)	0.0328 (18)	0.039 (2)	0.0001 (15)	0.0176 (18)	0.0045 (15)
N2	0.034 (2)	0.046 (2)	0.062 (3)	0.0034 (16)	0.025 (2)	0.0216 (18)
N3	0.031 (2)	0.047 (2)	0.040 (2)	−0.0043 (16)	0.0200 (19)	0.0012 (17)
C1	0.031 (2)	0.035 (2)	0.027 (2)	0.0002 (18)	0.014 (2)	0.0013 (17)
C2	0.044 (3)	0.041 (2)	0.039 (3)	0.005 (2)	0.022 (2)	0.0073 (19)
C3	0.031 (2)	0.035 (2)	0.031 (2)	0.0007 (19)	0.010 (2)	0.0015 (17)
C4	0.034 (2)	0.034 (2)	0.030 (2)	0.0019 (18)	0.017 (2)	0.0007 (18)
C5	0.035 (2)	0.037 (2)	0.040 (3)	−0.0008 (19)	0.019 (2)	0.000 (2)
C6	0.092 (4)	0.042 (3)	0.064 (3)	0.002 (2)	0.048 (3)	0.017 (2)
C7	0.028 (2)	0.044 (2)	0.046 (3)	0.0050 (19)	0.012 (2)	0.010 (2)
C8	0.028 (2)	0.049 (3)	0.051 (3)	−0.001 (2)	0.020 (2)	0.004 (2)
C9	0.084 (4)	0.096 (4)	0.045 (3)	0.004 (3)	0.017 (3)	−0.019 (3)

### Geometric parameters (Å, °)

**Table:**

S1—C2	1.710 (4)	N2—H2B	0.8600
S1—C3	1.748 (4)	C1—C2	1.349 (5)
O1—C5	1.318 (5)	C1—C4	1.449 (7)
O1—C6	1.451 (6)	C4—C5	1.509 (6)
O2—C5	1.184 (6)	C7—C8	1.505 (6)
O3—N3	1.431 (5)	C2—H2	0.9300
O3—C7	1.413 (4)	C6—H6A	0.9600
O4—C8	1.327 (6)	C6—H6B	0.9600
O4—C9	1.443 (6)	C6—H6C	0.9600
O5—C8	1.189 (5)	C7—H7A	0.9700
N1—C1	1.390 (5)	C7—H7B	0.9700
N1—C3	1.303 (7)	C9—H9A	0.9600
N2—C3	1.341 (6)	C9—H9B	0.9600
N3—C4	1.281 (5)	C9—H9C	0.9600
N2—H2A	0.8600		
			
S1···N1	2.583 (4)	N1···H2A^vii^	2.2200
S1···O5^i^	3.446 (4)	N2···H9A^viii^	2.9300
S1···N3^i^	3.486 (4)	N3···H6A^iii^	2.7700
S1···C8^i^	3.649 (6)	N3···H2A^vii^	2.6800
S1···C9^ii^	3.605 (5)	C1···C3^i^	3.430 (5)
S1···H9C^ii^	3.1100	C2···C6^v^	3.443 (6)
O1···O3	3.054 (4)	C3···C1^i^	3.430 (5)
O1···N3	3.111 (4)	C6···O3^vi^	3.408 (5)
O2···N3	3.257 (4)	C6···O4^iv^	3.385 (6)
O2···C6^iii^	3.337 (5)	C6···O2^vi^	3.337 (5)
O3···C6^iii^	3.408 (5)	C6···C2^ix^	3.443 (6)
O3···C8^iv^	3.231 (6)	C7···C7^iv^	3.530 (5)
O3···C7^iv^	3.284 (5)	C7···O3^iv^	3.284 (5)
O3···O5	2.747 (4)	C8···O3^iv^	3.231 (6)
O3···O1	3.054 (4)	C8···S1^i^	3.649 (6)
O4···C6^iv^	3.385 (6)	C9···S1^x^	3.605 (5)
O5···S1^i^	3.446 (4)	C2···H6B^v^	2.7800
O5···N3	3.100 (4)	C3···H9A^viii^	3.0700
O5···N2^i^	3.097 (5)	C4···H6A^iii^	2.9300
O5···O3	2.747 (4)	C5···H6A^iii^	3.0000
O1···H7A^iv^	2.8500	C5···H2	2.7500
O2···H6B	2.6600	H2···C5	2.7500
O2···H6A^iii^	2.7000	H2···O2^ix^	2.9100
O2···H6C	2.6000	H2A···N1^vii^	2.2200
O2···H2^v^	2.9100	H2A···N3^vii^	2.6800
O2···H6B^v^	2.8100	H2B···O5^i^	2.2700
O3···H7A^iv^	2.7400	H6A···O2^vi^	2.7000
O3···H6A^iii^	2.5300	H6A···O3^vi^	2.5300
O4···H6C^iv^	2.5100	H6A···N3^vi^	2.7700
O5···H9C	2.6500	H6A···C4^vi^	2.9300
O5···H7B^vi^	2.8300	H6A···C5^vi^	3.0000
O5···H9B	2.6100	H6B···O2	2.6600
O5···H2B^i^	2.2700	H6B···O2^ix^	2.8100
O5···H7A^iv^	2.8800	H6B···C2^ix^	2.7800
N1···S1	2.583 (4)	H6C···O2	2.6000
N1···N3	2.768 (6)	H6C···O4^iv^	2.5100
N1···N2^vii^	3.057 (5)	H7A···O1^iv^	2.8500
N2···N3^vii^	3.250 (5)	H7A···O3^iv^	2.7400
N2···O5^i^	3.097 (5)	H7A···O5^iv^	2.8800
N2···N1^vii^	3.057 (5)	H7B···O5^iii^	2.8300
N3···O5	3.100 (4)	H9A···N2^xi^	2.9300
N3···N2^vii^	3.250 (5)	H9A···C3^xi^	3.0700
N3···O1	3.111 (4)	H9B···O5	2.6100
N3···O2	3.257 (4)	H9C···O5	2.6500
N3···S1^i^	3.486 (4)	H9C···S1^x^	3.1100
N3···N1	2.768 (6)		
			
C2—S1—C3	88.9 (2)	O4—C8—O5	124.3 (4)
C5—O1—C6	115.8 (3)	O4—C8—C7	109.5 (3)
N3—O3—C7	107.2 (3)	O5—C8—C7	126.2 (4)
C8—O4—C9	116.6 (4)	S1—C2—H2	125.00
C1—N1—C3	109.6 (3)	C1—C2—H2	125.00
O3—N3—C4	110.6 (3)	O1—C6—H6A	109.00
C3—N2—H2B	120.00	O1—C6—H6B	109.00
H2A—N2—H2B	120.00	O1—C6—H6C	109.00
C3—N2—H2A	120.00	H6A—C6—H6B	109.00
N1—C1—C2	116.2 (4)	H6A—C6—H6C	110.00
N1—C1—C4	119.0 (3)	H6B—C6—H6C	109.00
C2—C1—C4	124.7 (3)	O3—C7—H7A	109.00
S1—C2—C1	110.4 (3)	O3—C7—H7B	109.00
N1—C3—N2	124.4 (3)	C8—C7—H7A	109.00
S1—C3—N2	120.7 (4)	C8—C7—H7B	109.00
S1—C3—N1	114.9 (3)	H7A—C7—H7B	108.00
N3—C4—C1	118.7 (4)	O4—C9—H9A	109.00
N3—C4—C5	123.9 (5)	O4—C9—H9B	109.00
C1—C4—C5	117.4 (4)	O4—C9—H9C	109.00
O2—C5—C4	122.8 (4)	H9A—C9—H9B	110.00
O1—C5—O2	125.6 (4)	H9A—C9—H9C	110.00
O1—C5—C4	111.6 (4)	H9B—C9—H9C	109.00
O3—C7—C8	111.1 (3)		
			
C3—S1—C2—C1	−0.1 (3)	O3—N3—C4—C5	0.9 (5)
C2—S1—C3—N1	0.4 (3)	N1—C1—C2—S1	−0.3 (4)
C2—S1—C3—N2	−178.8 (3)	C4—C1—C2—S1	−177.4 (3)
C6—O1—C5—O2	4.4 (8)	N1—C1—C4—N3	−19.6 (5)
C6—O1—C5—C4	−174.3 (4)	N1—C1—C4—C5	158.5 (3)
C7—O3—N3—C4	163.2 (3)	C2—C1—C4—N3	157.4 (4)
N3—O3—C7—C8	−76.2 (4)	C2—C1—C4—C5	−24.5 (5)
C9—O4—C8—O5	−4.5 (8)	N3—C4—C5—O1	−83.5 (5)
C9—O4—C8—C7	174.4 (4)	N3—C4—C5—O2	97.7 (6)
C3—N1—C1—C2	0.6 (4)	C1—C4—C5—O1	98.6 (5)
C3—N1—C1—C4	177.8 (3)	C1—C4—C5—O2	−80.2 (6)
C1—N1—C3—S1	−0.6 (4)	O3—C7—C8—O4	170.8 (4)
C1—N1—C3—N2	178.6 (3)	O3—C7—C8—O5	−10.3 (7)
O3—N3—C4—C1	178.8 (3)		

Symmetry codes: (i) −*x*, −*y*+1, −*z*+1; (ii) *x*−1, −*y*+3/2, *z*−1/2; (iii) *x*, *y*−1, *z*; (iv) −*x*+1, −*y*+1, −*z*+1; (v) −*x*, *y*−1/2, −*z*+1/2; (vi) *x*, *y*+1, *z*; (vii) −*x*, −*y*, −*z*+1; (viii) *x*−1, −*y*+1/2, *z*−1/2; (ix) −*x*, *y*+1/2, −*z*+1/2; (x) *x*+1, −*y*+3/2, *z*+1/2; (xi) *x*+1, −*y*+1/2, *z*+1/2.

### Hydrogen-bond geometry (Å, °)

**Table:**

*D*—H···*A*	*D*—H	H···*A*	*D*···*A*	*D*—H···*A*
N2—H2A···N1^vii^	0.86	2.22	3.057 (5)	163
N2—H2B···O5^i^	0.86	2.27	3.097 (5)	160
C6—H6A···O3^vi^	0.96	2.53	3.408 (5)	152
C6—H6C···O4^iv^	0.96	2.51	3.385 (6)	152

Symmetry codes: (vii) −*x*, −*y*, −*z*+1; (i) −*x*, −*y*+1, −*z*+1; (vi) *x*, *y*+1, *z*; (iv) −*x*+1, −*y*+1, −*z*+1.
